# Effectiveness of a mobile smoking cessation service in reaching elderly smokers and predictors of quitting

**DOI:** 10.1186/1471-2318-8-25

**Published:** 2008-10-06

**Authors:** Abu Saleh M Abdullah, Tai-Hing Lam, Steve KK Chan, Gabriel M Leung, Iris Chi, Winnie WN Ho, Sophia SC Chan

**Affiliations:** 1 Department of International Health, Boston University School of Public Health, Boston, Massachusetts, USA; 2 Department of Community Medicine, School of Public Health, Li Ka Shing Faculty of Medicine, The University of Hong Kong, Hong Kong, PR China; 3 School of Social Work, University of Southern California, Los Angela, USA; 4 Department of Nursing Studies, Li Ka Sing Faculty of Medicine, The University of Hong Kong, Hong Kong, PR China; 5 Department of Social Work, Hong Kong Baptist University, Hong Kong, PR China

## Abstract

**Background:**

Different smoking cessation programmes have been developed in the last decade but utilization by the elderly is low. We evaluated a pilot mobile smoking cessation service for the Chinese elderly in Hong Kong and identified predictors of quitting.

**Methods:**

The Mobile Smoking Cessation Programme (MSCP) targeted elderly smokers (aged 60 or above) and provided service in a place that was convenient to the elderly. Trained counsellors provided individual counselling and 4 week's free supply of nicotine replacement therapy (NRT). Follow up was arranged at 1 month by face-to-face and at 3 and 6 months by telephone plus urinary cotinine validation. A structured record sheet was used for data collection. The service was evaluated in terms of process, outcome and cost.

**Results:**

102 governmental and non-governmental social service units and private residential homes for the elderly participated in the MSCP. We held 90 health talks with 3266 elderly (1140 smokers and 2126 non-smokers) attended. Of the 1140 smokers, 365 (32%) received intensive smoking cessation service. By intention-to-treat, the validated 7 day point prevalence quit rate was 20.3% (95% confidence interval: 16.2%–24.8%). Smoking less than 11 cigarettes per day and being adherent to NRT for 4 weeks or more were significant predictors of quitting. The average cost per contact was US$54 (smokers only); per smoker with counselling: US$168; per self-reported quitter: US$594; and per cotinine validated quitter: US$827.

**Conclusion:**

This mobile smoking cessation programme was acceptable to elderly Chinese smokers, with quit rate comparable to other comprehensive programmes in the West. A mobile clinic is a promising model to reach the elderly and probably other hard to reach smokers.

## Background

Cigarette smoking is the leading cause of premature mortality among older persons in Hong Kong [1,2] and elsewhere.[3] Many common diseases among older people are caused by tobacco use.[4,5] The World Bank has estimated that five hundred million people alive today will eventually be killed by tobacco.[6] Worldwide trends in mortality attributable to smoking will increase in both older men and women.[7]

The prevalence of cigarette smoking was 14% in Hong Kong people aged over 60 in the 1998 General Household Survey and there were a total of 129,600 older smokers at that time [8]. A higher prevalence of current smoking was reported in studies conducted among older people aged 60 and over by the Hong Kong Society for the Aged (19%) [9] and by the University of Hong Kong (19%) [10]. In Hong Kong, there has been a lack of smoking cessation services and there is no evidence whether such services could help older people to quit smoking. Nevertheless, about 15% of smokers aged 60 and older wanted to quit within the next 6 months [10] and evidence elsewhere shows that older smokers are more likely to be successful in quitting attempts than smokers aged 35–64.[11] This paper reports the acceptance and benefits of smoking cessation services among older smokers in Hong Kong.

Although a variety of smoking cessation programmes have been developed in the last decade, utilization by the elderly is low.[12] A frequently cited reason is inconvenience in reaching the services, [13] because the service locations are not near to their living environment. On the other hand, many elderly people live alone or in elderly homes and traveling to a smoking cessation clinic far away is not practicable. A more accessible service should encourage more people to utilize the service and benefit from it. Mobile clinical service was useful in reaching the hard to reach population in other settings.[14] However, we found no such reports in the literature that targeted elderly smokers with a mobile smoking cessation service. We examined the effectiveness of a mobile smoking cessation service in reaching elderly Chinese smokers in Hong Kong and identified predictors of quitting. We aimed to answer four specific questions: (1) Would Chinese elderly smokers participate in a mobile smoking cessation programme (MSCP)? (2) Is the programme effective in promoting smoking cessation among elderly Chinese smokers? (3) What are the predictors of quitting among the Chinese elderly? (4) What are the costs of the programme?

## Methods

### Mobile smoking cessation programme (MSCP)

The Departments of Community Medicine and Nursing Studies and School of Public Health of the University of Hong Kong with funding from the Elderly Commission, Government of the Hong Kong Special Administration Region developed a Mobile Smoking Cessation Programme (MSCP) to reach elderly smokers (aged 60 or above). The MSCP started in November 2002 and continued till September 2004. The mobile team included a coordinator and 3 trained smoking cessation counsellors. These counsellors were registered nurses and had completed satisfactorily a smoking cessation counselling training programme with assessment by written and practical examinations. The mobile team was supported by a project director specialized in smoking cessation. The MSCP included health talks, assessment of clients' smoking status and nicotine dependence level, provision of individually tailored behavioural counselling, prescription of nicotine replacement therapy, NRT (patch only), and arrangements for follow up (telephone and on-site). We recommended subjects to use NRT for 8 weeks and gave out free supply for the first 4 weeks. We followed social cognitive theory (SCT) to design the intervention of the program. SCT explains why a behavior occurs positing that there is a three-way reciprocal interaction between the environment, the individual and a behavior [15]. The SCT has been successfully applied in several clinical and community based studies of smoking cessation [16,17].

### Target population and recruitment

The eligible subjects were current smokers who were attending 102 social service units or private residential homes (both Government and non-Government) throughout Hong Kong to receive health services or elderly care. All these service units were specialized in service provision for the elderly. We invited a social worker, if available, in each of these centres to act as our contact person. These social workers were trained by us on basic smoking cessation skills, the details of which were described elsewhere.[18,19] The respective social worker from each of these centres identified elderly smokers within their service areas and confirmed a date for the visit of the MSCP team. Elderly non-smokers and family members of elderly smokers who were interested to know about smoking and health issues were also encouraged to attend the health talks, but were not included in the analysis. Most of the private homes did not have social workers and some clients were referred by other staff members.

The health talks were organized in the premises of the social service centres. Each health talk continued for about an hour. The nurse counsellors from the mobile team delivered pre-designed talks (about 30 minutes) and discussed on different aspects of smoking cessation. The content included the harms of tobacco use (both active and passive smoking), benefits of quitting smoking and tips for quitting. Informal discussion, experience sharing, and a question and answer session were conducted during the second half of the talk, including brief information about the MSCP.

**Figure 1:**
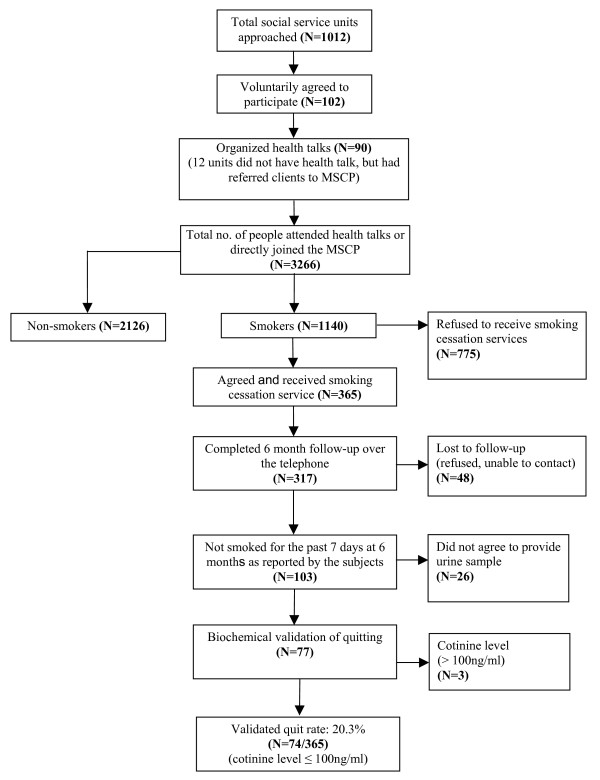
Flow of subject recruitment in the Mobile Smoking Cessation Programme (MSCP).

After the health talks, all smokers were asked to enrol for an intensive smoking cessation service, including cognitive-behavoural stage matched counselling and use of NRT, which lasted for about half an hour, provision for free NRT supply for 4 weeks and follow up arrangements. Those who consented to participate were included in the programme (Figure 1). Ethical approval for this study was obtained from the Ethics Committee of the Faculty of Medicine, the University of Hong Kong.

### Data collection

A structured questionnaire was used to collect data at baseline and at 1, 3 and 6 months. Details of the questionnaire were described elsewhere.[20,21] Briefly, the questionnaire included demographic information, smoking and quitting history, nicotine dependence level, and perceived motivation (self efficacy rating), confidence and difficulty of quitting smoking (perceived barriers). The client's satisfaction towards different aspects of MSCP was assessed at 3 month follow up, including counselling received in the centre and/or over the telephone, and follow up arrangements. Counsellors' satisfaction was assessed from the meeting notes, every 2 months. We asked the counsellors questions about the process of service delivery, operational guidelines, work load, availability of support and job satisfaction. Satisfaction was rated on a four-point scale (*very satisfactory, satisfactory, unsatisfactory and very unsatisfactory)*.

### Follow up assessment

Follow up assessment and relapse prevention counselling was carried out at 1 month post-counselling by face-to-face and at 3 and 6 months by telephone for all who attended the MSCP. Both face-to-face and telephone follow up lasted for an average of 20 minutes. We also made a follow up call (lasting for 2–5 minutes) at 1 week to assess whether the elderly were having any problem with NRT use and encourage further use. The nurse counsellors carried out follow up interviews. At 6 months, those who stopped smoking (not smoking for 7 days or more preceding the follow up interview) were invited to attend the nearest social service unit for biochemical validation (by measuring urinary cotinine level). For those who could not attend for validation, our research assistant visited the subjects to collect urine samples.

### The evaluation

The programme was evaluated in terms of *process, outcome *and *cost*. The *process *evaluation comprised documentation of comments or suggestions from the nurse counsellors and other members of the MSCP team, and satisfaction ratings of the subjects regarding counselling and follow up arrangements. The main *outcome *evaluation was based on the validated 7 day point prevalence quit rate at 6 months, and we also reported several other quitting outcomes as secondary outcomes. Other process outcomes included number of MSCP organized, number of elderly members attended the health talks, number of elderly smokers who had received intensive smoking cessation service (counselling and/or NRT) and contributions to smoking cessation research in Hong Kong and elsewhere. We calculated all the relevant costs (staff salary, stationery, NRT, travel for the mobile team, and urine cotinine tests) and divided the total costs by the total number of attendees/quitters and compared them with other relevant programmes.

### Data analysis

Data were analyzed using SPSS for Windows, version 10.0. The baseline characteristics of clients were described. The prevalence of quitting in the MSCP was compared with those of studies elsewhere. The characteristics of quitters and non-quitters were compared by chi-square test. The variables which were significant in the bivariate analysis were tested by forward stepwise logistic regression modeling to identify predictors for quitting and to estimate adjusted odds ratios and 95% confidence intervals (CI).

Quitting smoking was defined as not smoking any cigarettes during the past 7 days at 6 month follow up as reported by the subjects and confirmed by urine cotinine validation (7 day *point prevalence validated quit rate*). All subjects who could not be contacted at 6 month follow up and those who failed the validation test (a urinary cotinine level > 100 ng/ml) [22] were considered as smokers (i.e. had no change from baseline) based on intention-to-treat analysis (a conservative approach). As secondary outcomes, we also measured 7 day point prevalence quit rate at 6 months without validation (defined as not smoking during the 7 days preceding the 6 month follow up), 24 hour point prevalence quit rate at 6 months without validation (defined as not smoking during the 24 hours preceding the 6 month follow up), continuous abstinence rate (abstinence from tobacco smoking continuously for the whole period prior to the interview at 6 months) [23] and reduction in smoking rate (reduction of the amount smoked by at least 50% at 6 month follow up).[24]

## Results

### Utilisation and process evaluation

During the study period, we contacted a total of 1012 governmental and non-governmental social service units and private residential homes for the elderly, and 102 participated in the MSCP. We organized 90 health talks (12 units did not require health talk but recruited smokers to receive our intensive counselling) with 1140 smokers attended. Of the 1140 smokers, 365 (32%) agreed to receive our smoking cessation service. The demographic, lifestyle and quitting characteristics of these subjects are shown in Table 1.

**Table 1:** Demographic, lifestyle, smoking and quitting related factors of 365 smokers to the mobile smoking cessation programme (MSCP) participants (n = 365)

***Characteristics***	%
**Demographics:**	
*Gender*	
Male	71
Female	29
*Occupational status*	
Retired	86
Unemployed	6
Employed	1
Homemakers	7
*Age*	
60–69	21
70–79	41
80 or above	38
*Educational attainment*	
No formal education	39
Primary school	46
Secondary or above	15
*Marital Status*	
Single	15
Married	44
Divorced, separated and widowed	41
**Tobacco use related:**	
*Daily cigarette consumption*	
≤ 10	70
> 10	30
*Age started smoking*	
Under 20	62
20 or above	38
*Nicotine Dependency level *^*a*^	
Mild	65
Moderate	20
Severe	15
*Number of other smokers in household*	
Nil	71
1 or more	29
*Smoking status of spouse*	
No spouse/spouse not smoker	90
Spouse is smoker	10
**Quitting History:**	
*Number of previous quitting attempt(s)*	
Nil	41
1 attempt or more	59
*Length of abstinence in the last quitting attempt*	
Less than a day or not at all	5
> = 1 day	95
**NRT related:**	
*Use of NRT for at least 1 day*^*b*^	
No	23
Yes	77
*Adherence to use NRT for 4 weeks or more *^*b*, *c*^	
No	62
Yes	38
**Other factors:**	
*Perceived importance on quitting (mean score = 73)*^*d*^	
Less important (< mean)	48
More important (> = mean)	52
*Perceived difficulties on quitting (mean score = 55)*^*e*^	
Less difficult (< mean)	53
More difficult (> = mean)	47
*Perceived confidence on quitting (mean score = 67)*^*f*^	
Less confident (< mean)	42
More confident (> = mean)	58
*Alcohol consumption*^*g*^	
Regular user and occasional users	18
Never or rarely drink	82

Note: Total percentage may be more or less than 100 due to rounding of the figures.

^a ^Nicotine dependency level was measured by Fagerstrom scale, then further divided into 3 levels: low (score 0–3), moderate (score 4–8) and severe (score 6–10).; n = 354, 11 missing.

^b^Included only those who were given NRT (n = 255).

^c^Subjects who reported using nicotine replacement therapy (NRT) for at least 4 weeks during the 3 month follow up was defined as adherent to NRT use.

^d^Total n = 352, 13 missing

^e^Total n = 356, 9 missing

^f^Total n = 355, 10 missing

^g^Total n = 345, 20 missing

#### Satisfaction

More than 90% of the respondents were satisfied with the counselling service and follow up arrangements. Eighty five percent of the subjects would probably or definitely recommend this programme to other smokers. However, 80% of the subjects were asking for free full course of NRT, which was not possible as our funding could only support 4 week free supply.

Structured interview was also conducted with all the four counsellors and the coordinator, which showed that all the staff members were satisfied with the counselling process, supervisory support and workload. The main barrier was the travel time and communication difficulties with the elderly. Repeated reminders were necessary as many elderly often forgot about their follow up arrangements. No complaints about the programme and no other major difficulties were encountered.

### Outcome evaluation

#### Primary outcome (urine cotinine validated quit rate)

At 6 month follow up, 48/365 (13%) subjects could not be reached (left Hong Kong, telephone number changed or refused to talk) and 103 subjects reported that they did not smoke in the 7 days preceding the 6 month follow up (Table 2). All the 103 self-reported quitters were invited for biochemical validation with urine cotinine test (Nicalert test) and 77 gave urine samples. Three of these had a cotinine level of > 100 ng/ml and were considered as smokers. By intention-to-treat analysis, the validated (cotinine level = < 100 ng/ml) quit rate was 20.3% (74/365) (95% confidence interval, CI: 16.2%–24.8%).

**Table 2:** Quitting outcome of 365 smokers in the MSCP at 6 month follow up, by intention to treat

**Quit rates**	**N**	**% (95% confidence interval)**
***Primary outcome:***		
Biochemically (urine cotinine) validated quit rate	74	20.3 (16.2 – 24.8)

***Secondary outcomes (self-reported)***		
7 day point prevalence quit rate	103	28.2 (23.6–32.8)
24 hours point prevalence quit rate	110	30.1(25.4–34.8)
Continuous abstinence quit rate	91	24.9 (20.5–29.4)
Had not quit but had reduced smoking by at least 50% from the baseline level	94	25.8(21.3 – 30.2)

#### Secondary outcomes

At 6 month follow up, by intention-to-treat analysis, of the 365 subjects, 28.2% did not smoke any cigarettes during the 7 days prior to the interview (*7 day point prevalence*), 30.1% did not smoke any cigarettes during the 24 hours prior to the interview (*24 hour point prevalence*), and 24.9% did not smoke any cigarettes during the six months prior to the interview (6 *month continuous abstinence)*, and25.8% reported that they did not quit but had reduced daily smoking by at least 50% (*reduction rate*) (Table 2).

### Factors associated with quitting at 6 months

With the inclusion of those who did not return for follow-up as non-quitters, we carried out bi-variate analysis of all the eighteen variables in Table 1 to identify factors associated with quitting. Nine factors were significantly associated with quitting: smoking less than 11 cigarettes per day, having made one or more serious quitting attempts in the past, being moderately or mildly dependent on nicotine, quitting for at least a day in the last quitting attempt, using NRT for at least a day in the present quitting attempt, being adherent to NRT use for 4 weeks or more, perceiving more importance and confidence on quitting, and having an exhaled carbon monoxide level of below the mean (< 10 ppm) at the first visit. Stepwise logistic regression modelling on these nine factors showed that smoking less than 11 cigarettes per day and being adherent to NRT use for 4 weeks or more were the two significant independent predictors of quitting (Table 3).

**Table 3:** Final logistic regression (forward stepwise) model to predict successful quitting at 6 month follow up

**Independent variables**	**OR (95% CI)**	**P value**
Smoking less than 11 cigarettes per day	2.63 (1.37–5.06)	< 0.01
Adhered to NRT use for 4 weeks or more	3.57 (1.95–6.55)	< 0.001

Note: OR = adjusted odds ratio; CI = Confidence interval

### Costs of the MSCP

The cost of the MSCP included mainly operation cost (staff salary and stationery), cost of equipment and NRT and travel cost for the mobile team. Some hidden costs such as time spent by the programme director and other members of the project team (who were involved mainly in the planning, monitoring, evaluation and research), and client costs (travel time, travel cost, cost to other family members) were not included. The total expenditure for the operation of MSCP was US$ 61,162 (Table 4). The MSCP had 1140 smoker subjects for health talks and 365 smokers for smoking cessation service. At 6 month follow up, 103 smokers did not smoke any cigarettes during the past 7 days. 74/77 of the self-reported quitters who had cotinine validation were confirmed as quitters. Therefore, the cost per contact was US$53.65 (smokers only); cost per smoker with counselling was US$167.57; and cost per self-reported quitter was US$593.81 and per cotinine validated quitter, US$826.54.

**Table 4:** Costs for the Mobile Smoking Cessation Programme (MSCP)

**Costs**	**HK$**
Fixed capital costs	Not costed
Stationary and equipment	1,220
	
*MSCP service costs*	
Salary of part-time smoking cessation counsellors	328,569
Salary of other part-time supporting staff	18,671
	
*NRT costs*	
Costs for 4 week free supply (50% discounted price)	109,223
	
*Biochemical validation costs*	
Cost for urine cotinine test	7,294
	
*General expenses*	
Transportation	8,622
Photocopies	3,465
Hidden cost (Time spent by the programme director and other members of the project team)	Not costed

**Total cost (in HK$)**	**477,064**

**Total cost (in US$)**	**61,162** (US$47,159 excluding NRT costs)

Note: US$1 = HK$7.8

Cost per smoker contact = US$61162/1140 = US$53.65

Cost per subject with intensive counselling = US$61162/365 = US$167.57

Cost per successful self-reported quitter (intention to quit) = US$61162/103 = US$593.81 (US$457.82, excluding NRT costs)

Cost per validated quitter (intention to quit) = US$61162/74 = US$826.54 (US$637.18, excluding NRT costs)

## Discussion

Our experience and findings from the mobile smoking cessation programme (MSCP) suggest feasibility and acceptance of this outreach programme among the Chinese elderly. Although the project was not designed as a controlled experiment, the results suggest that mobile smoking cessation programmes can be effective for the hard to reach population such as the elderly, provided that the needs and difficulties of the targeted population are addressed. While a community-based study in the United States reported that smokers aged 65 and older were least likely to use a smoking cessation programme, [8] our mobile service was reasonably accepted as reflected from clients' participation and enthusiasm. It was convenient for many elderly clients who could not travel to receive services far away. Few community smoking cessation projects used cotinine validation for evaluation of quitting outcomes. Our study has the strengths in the use of cotinine validation and the high percentage (75%) of acceptance of the validation. It is worth mentioning that our cessation service was not able reach all the elderly smokers in the 102 participating social service units. Although we trained social workers in each of these units to refer smokers to our program, we did not conduct any baseline survey of all the residence in the studies social service units nor did record data about what proportion of the smokers actually attended the program. However, based on our exploratory estimate, we assume that our program reached at least 70% of the smoker population in these social service units.

Our service (individual counselling and 4 week free supply of NRT) resulted in a self-reported 6-month point prevalence quit rate (by intention-to-treat) of 28%, which was comparable with the one year point prevalence self-reported quit rate (27%) among Chinese adult smokers who attended the Hong Kong Smoking Cessation Health Centre, [25] and higher than the 6 month point prevalence self-reported quit rate (14.4%) in clinic based smoking cessation services in New Zealand [26] and the United States (22%).[27] Our quit rate was also comparable to the 7-day point prevalence self-reported quit rate (29%) among American elderly (aged 65 to 74 years) who also used nicotine patch for an average of 5 weeks.[28] While our quit rates seems better than the above studies abroad [26-28], few clarifications worth noting. Our subject included a higher proportion (70%) of those who were light smokers (smoked less than 10 cigarettes daily), however, based on our review of these studies [26,27] papers, a higher proportion of subjects in other studies [26-28] were moderate or heavy smokers. The average daily consumption of our subject was 10 cigarettes per day compared to the mean number of 25.4 cigarettes per day among American smokers [27] and a median of 20 (range 1–85) among smoker in New Zealand [26].

The cost per self-reported quitter (US$458, excluding cost for NRT) was 35% higher than that in the Hong Kong Smoking Cessation Health Centre, which was the first such clinic in Hong Kong (US$339, excluding cost for NRT).[22] The higher cost was mainly due to the mobile nature of the service, longer duration of counselling needed for elderly clients, and the additional costs for cotinine validation.

We found that being a light smoker (smoking less than 11 cigarettes per day) and using NRT for four weeks or more were significant independent predictors of quitting. This suggests that heavy smokers might need to be targeted with more intensive programmes.[29] Efforts to increase NRT adherence are also needed to improve quit rates.[20] Provision of free NRT supply for a longer duration (full course, 8 weeks) is suggested for those who want to use but cannot afford.

A major limitation of the study is the lack of a control group to compare the elderly who participated in our MSCP with those who did not. However, our validated quit rate of 20.3% is about two times the natural self-reported quit rate (10.0%) among the elderly aged 65 or above in the US general population.[30] Moreover, participation in the study was voluntary and this might have resulted in the recruitment of more motivated smokers from the general population. On the other hand, motivation of clients to a mobile service would be lower than that among those who travel to a clinic further away. Some of our smokers could have attended the service due to the pressure and/or encouragement from other family members or social workers from the social service units, and the prohibition of smoking inside most units. It is also possible that free counselling service and offer of 4-week's supply of NRT free of charge encouraged many smokers to attend the program.

This study has important public health implications. First, to the best of our knowledge, this is the first study to report promotion of smoking cessation programme through mobile service targetting the elderly smokers, and our experiences and results should be important for other smoking cessation service providers. Second, the low cost of the programme suggests that a mobile service could be promoted to attract more smokers in addition to the elderly. A timetable convenient to the target clients is needed and should be publicized through the health care facilities, elderly homes and other health centres. Health care and social service providers could be motivated to identify older smokers and, if no smoking cessation services are provided in their premises, could refer them to the appropriate mobile service scheduled nearer to their residents or clients. Mass media promotional activities would increase the coverage but the cost would be high and local publicity should be more affordable. The setting up of a smoking cessation service can provide a golden opportunity for publicity. For example, we held two exhibitions, which attracted about 800 people to visit our booths and collect self-help materials. Integration of the smoking cessation service with other existing mobile service (if any) can reach more clients with shared costs. Finally, it would be useful to test the effectiveness and cost effectiveness of mobile smoking cessation service for other vulnerable population groups (such as pregnant women and young people).

## Conclusion

We conclude that a mobile smoking cessation program is a feasible approach to reach elderly smokers. The quit rate is comparable to other comprehensive programs in the West. We identified several predictors of quitting smoking through the mobile program which could guide the future service provision. A mobile clinic is a promising model to reach the elderly and probably other hard to reach smokers.

## Competing interests

The authors declare that they have no competing interests.

## Authors' contributions

ASMA and THL originated the study, developed study protocol and supervised all aspects of its implementation. ASMA performed data analysis and drafted the manuscript. SKC and WNH assisted in data collection and in the analysis. THL provided in-depth comments in the earlier draft of the manuscript. GML, IC and SCC contributed to the interpretation of data and revising the final draft of the manuscript. All authors reviewed drafts of the manuscript and approved the version to be published.

## Pre-publication history

The pre-publication history for this paper can be accessed here:


